# A rare dislocation: an open isolated palmar dislocation of the head of the fifth metacarpal bone without fracture

**DOI:** 10.1080/23320885.2021.1956934

**Published:** 2021-07-26

**Authors:** Tinatin Natroshvili, Rombout Molenaar

**Affiliations:** Department of Plastic Surgery, Treant Health Care Group: Hospital Location Bethesda, Hoogeveen, The Netherlands

**Keywords:** Hand joints, anatomy, metacarpal bone, dislocation

## Abstract

We present an open and isolated palmar dislocation of the head of the fifth metacarpal bone without fracture. The diagnosis, which was initially made based on the X-rays, was confirmed during the operation. The patient was satisfactorily treated with open reduction, Kirschner wires fixation and casting followed with hand physiotherapy.

## Introduction

The volar plate and collateral ligaments prevent MCP joints (metacarpophalangeal joints) of dislocation due to protection against hyperextension, radial and ulnar deviation. The most common dislocation of MCP joint is a dorsal dislocation [1] and the most common digits dislocated in MCP joint are index and thumb. Isolated palmar metacarpophalangeal joint dislocation of the head of the fifth finger without a fracture is very rare.

Therefore, the purpose of this report is to present a patient with an isolated palmar dislocation of the head of the fifth metacarpal bone without a fracture, satisfactorily treated with open reduction, Kirschner wires fixation, casting, and followed with hand physiotherapy and hand occupational therapy.

## Case report

**Figure 1.**
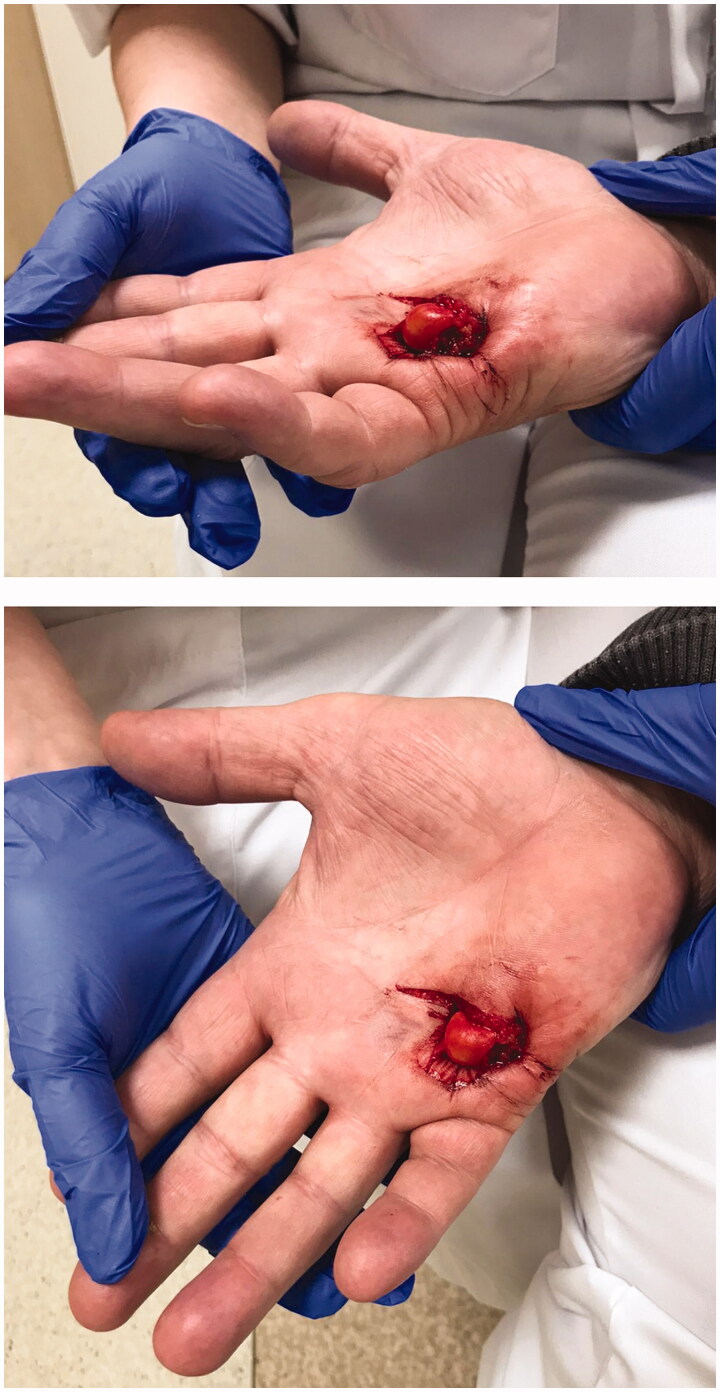
Photograph of the right hand during the examination at the ED.

A 62-year-old right-handed male presented to the Emergency Department (ED) with palmar protrusion of the head of the fifth metacarpal bone through the skin following a fall with injury to the right hand, Figure 1.

On examination, patient had besides an open wound with palmar protrusion the fifth metacarpal bone through the skin, pain around the wound. However, capillary refill and sensation were normal, which suggested an intact neurovascular bundle of the fifth finger. Further, normal flexion of distal interphalangeal joint and the proximal interphalangeal joint was observed. Wrist and other movements were normal. The patient had no previous history of trauma to the right hand.

**Figure 2.**
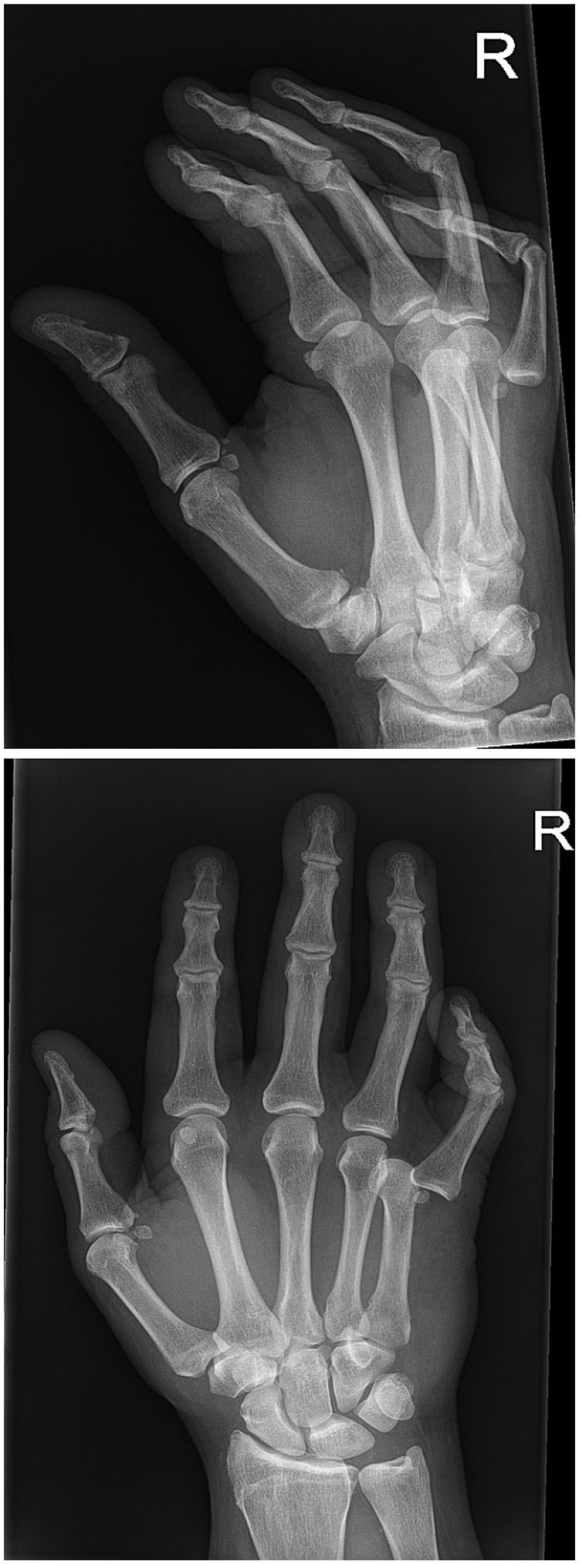
X-ray of the right hand during the examination at the ED.

Standard radiographs were obtained which revealed isolated palmar dislocation of the head of the fifth metacarpal bone without any other associated injury or fracture, Figure 2.

An attempt to perform a closed reduction under local anesthesia was unsuccessful. The patient was admitted to undergo an open reduction under regional anesthesia the same day.

**Figure 3.**
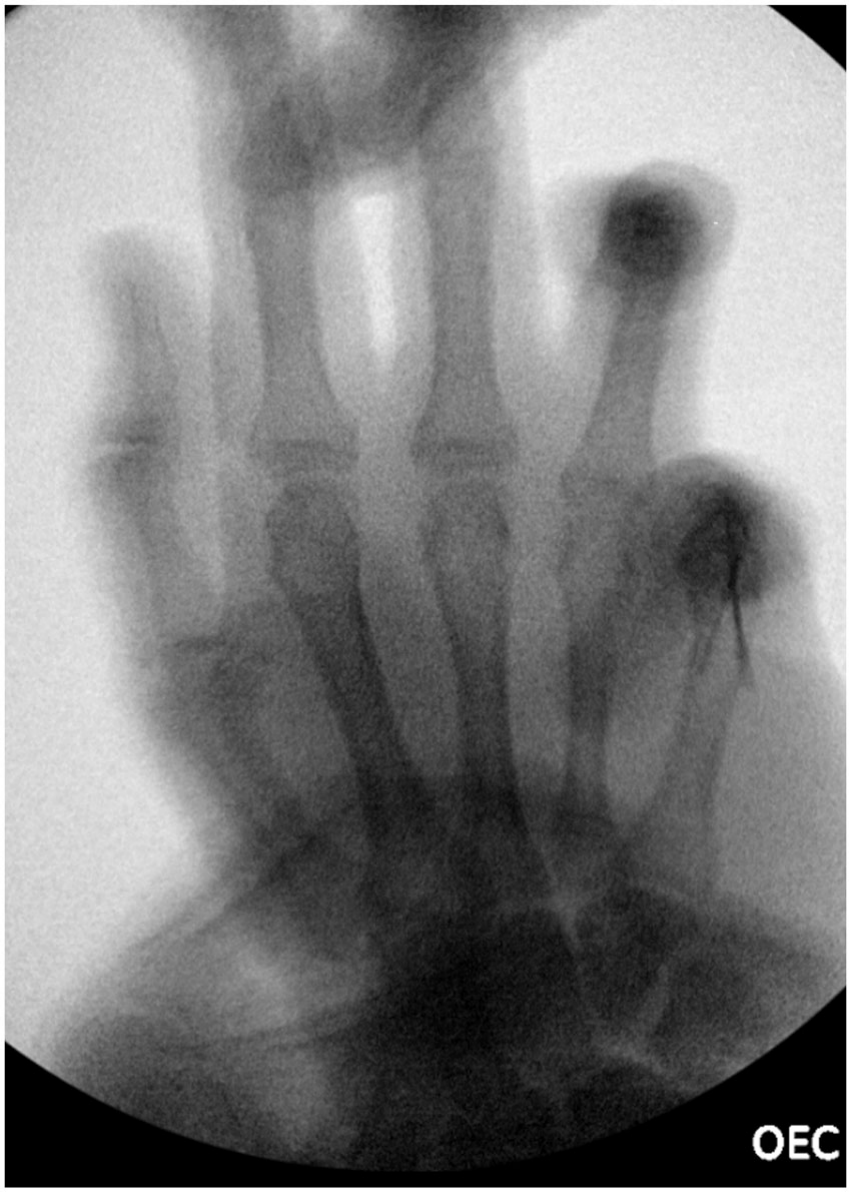
X-ray of the right-hand showing K-wire fixation.

**Figure 4.**
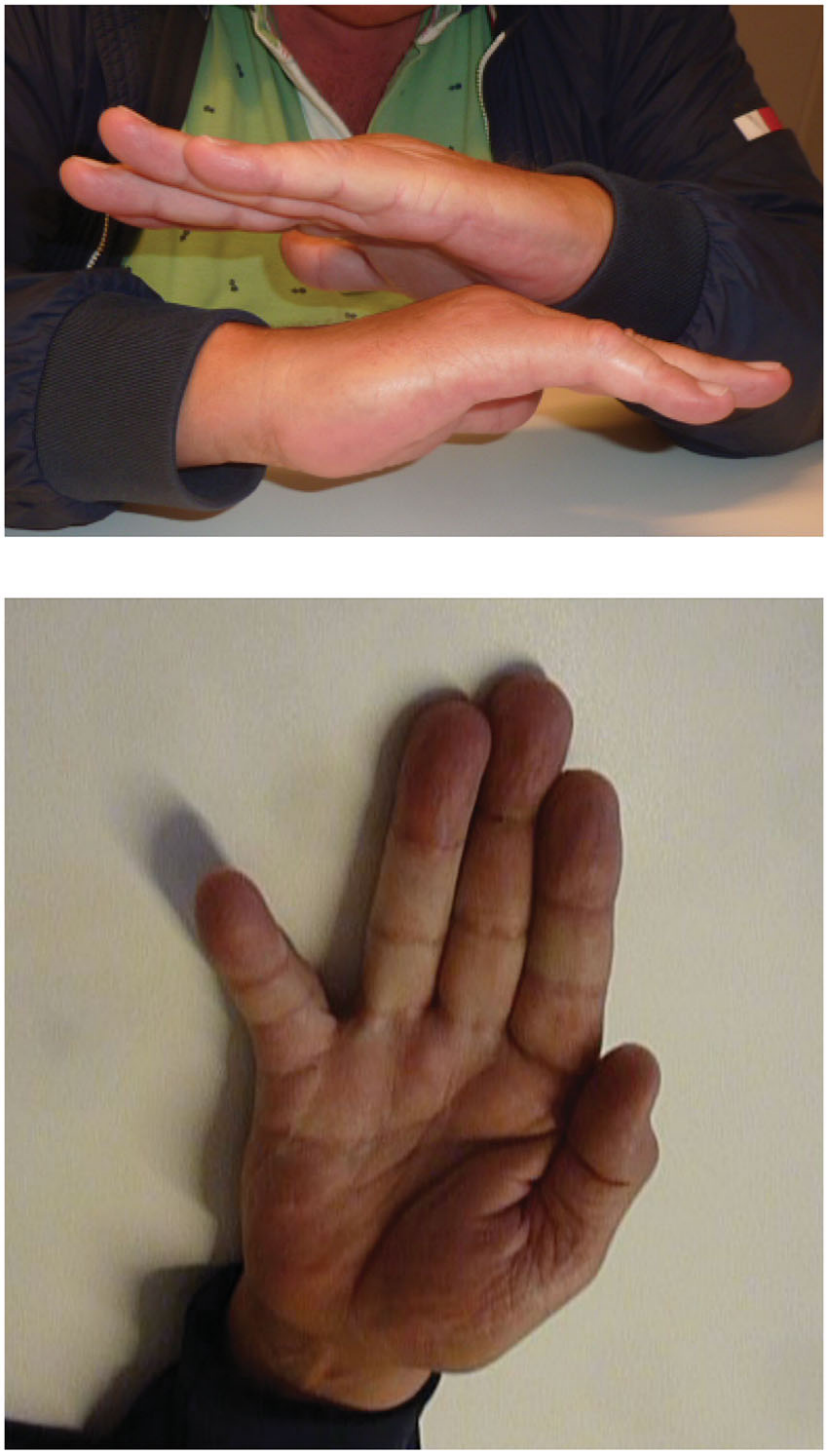
Photograph of the right hand at follow-up (6.5 months).

**Figure 5.**
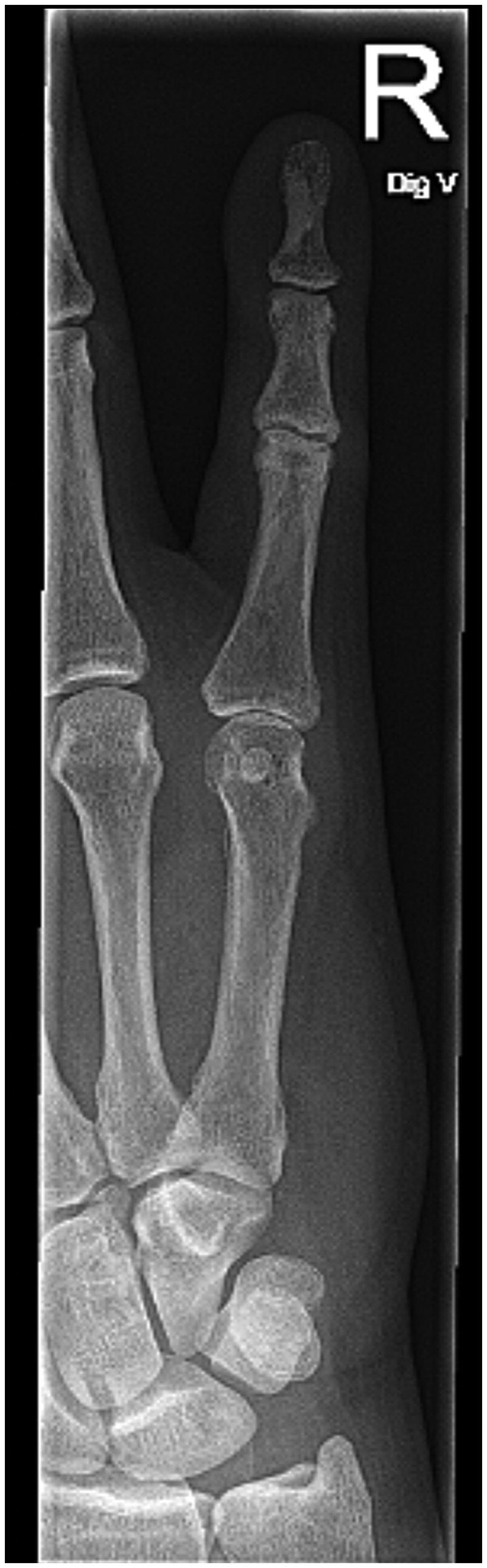
X ray of the right hand at follow-up (6.5 months).

Extending the wound, we observed a dislocation of flexor digitorum superficialis tendon (FDS) and flexor digitorum profundus tendon (FDP) to radio dorsal from the head of the fifth metacarpal bone. After replacing tendons to palmar, dislocation dissolved. Neither reconstruction of the volar plate nor exploration of the intermetacarpal ligaments were performed. After achieving hemostasis, the wound was closed with Ethilon 5-0. Internal stabilization with Kirschner- wires through MCP joint in 90 degrees of flexion was performed, see Figure 3. The below-elbow splint with MCP joints in 90 degrees flexion was applied. The perioperative period was uneventful. The sutures and the K-wires were removed on 14th postoperative day and 3 weeks after surgery. Patient received 14 sessions of hand physiotherapy and hand occupational therapy during four consecutive months for active and passive mobilization of fingers and wrist. Seven months following injury the patient regained full range of flexion DIP, PIP and IP- joints of fifth digit and full stability of the finger. However, when the hand was hold in anatomical position fifth digit showed abduction and limited extension, see Figures 4 and 5 for X-ray performed at the follow up. This did not disturbed patient in daily activities, and he was satisfied with the results.

## Discussion

An isolated open palmar dislocation of the head of the fifth metacarpal bone without any fracture is rare. Reposition of this type of dislocations can be practically impossible due to volar plate, collateral ligament, tendinous juncture connecting the fourth and fifth extensor digitorum communis tendons, sesamoid bone, or as observed in our case luxation of FDS and FDP to dorsoradial to the head of the metacarpal bone [2]. Considering the information obtained duringthe operation, it is not a surprise, that closed reposition at the ED wasn’t successful. On the other hand, the open reposition and fixation showed, in our case, a satisfactory result.

The volar plate wasn’t reconstructed during the surgery, because it required a total exposure of the joint capsule. During the operation, some stability of the joint was noticed due to the surrounding structures, and given the obtained results we can assume that during the healing process affected joint developed more stability due to the scar tissue and the formation of adhesions.

To the best of our knowledge, this is the first published case of open isolated palmar dislocation of the head of the fifth metacarpal bone without fracture.

In our opinion, a closed reduction of rare palmar MCP joint dislocations have little chance of success due to volar plate, collateral ligament, tendinous juncture connecting the fourth and fifth extensor digitorum communis tendons, sesamoid bone, or as observed in our case luxation of FDS and FDP to dorsoradial to the head of the metacarpal bone. The open reposition and fixation showed, in our case, good results after sessions of hand physiotherapy and hand occupational therapy.
